# National survey of do not attempt resuscitation decisions on out-of-hospital cardiac arrest in China

**DOI:** 10.1186/s12873-022-00581-0

**Published:** 2022-02-11

**Authors:** Sijia Tian, Shengmei Niu, Luxi Zhang, Huixin Lian, Ming Zhou, Xuejiao Zhang, Xuqin Kang, JinJun Zhang

**Affiliations:** Beijing Emergency Medical Center, Beijing, 100031 China

**Keywords:** National survey, Do not attempt resuscitation, OHCA, Emergency medical services

## Abstract

**Background:**

To investigate and understand the determinants of decisions not to attempt resuscitation following out-of-hospital cardiac arrest, to contribute to establishing rules that are appropriate to China.

**Methods:**

We recruited participants through directors of emergency medical services across China. A 28-question web survey was available between February 5 and March 6, 2021 that targeted demographic information and views on emergency work and cardiopulmonary resuscitation. Each question was assigned a value between 1 and 7 based on the level of importance from low to high. T-tests, one-way analysis of variance, and Kruskal–Wallis H-tests were used to compare continuous variables. Binary logistic regression analysis was used to identify factors influencing when people considered it suitable to initiate cardiopulmonary resuscitation.

**Results:**

The study involved 4289 participants from 31 provinces, autonomous regions and municipalities in mainland China, of whom 52.8% were male. The top three reasons for not attempting cardiopulmonary resuscitation were decomposition/hypostasis/rigor mortis (6.39 ± 1.44 points), massive injury (4.57 ± 2.08 points) and family members’ preference (4.35 ± 1.98 points). In total, 2761 (64.4%) thought emergency services should not attempt cardiopulmonary resuscitation when cardiac arrest had happened more than 30 min before, and there had been no bystander cardiopulmonary resuscitation. Gender (OR 1.233, *p* = 0.002), religion (OR 1.147, *p* = 0.046), level (OR 0.903, *p* = 0.028) or classification of city (OR 0.920, *p* = 0.049), years of work experience (OR 0.884, *p* = 0.004), and major (OR 1.032, *p* = 0.044) all influenced how long after cardiac arrest was considered suitable for initiating cardiopulmonary resuscitation.

**Conclusions:**

Chinese emergency physicians have different perceptions of when not to attempt resuscitation to those practicing elsewhere. The existing guidelines for resuscitation are not suitable for China, and China-specific guidelines need to be established.

**Supplementary Information:**

The online version contains supplementary material available at 10.1186/s12873-022-00581-0.

## Background

“Do not attempt resuscitation” and “do not attempt cardiopulmonary resuscitation” decisions allow cardiopulmonary resuscitation to be withheld where it stands little or no chance of success, when the risks outweigh the benefit or if someone asks not to receive cardiopulmonary resuscitation [1]. Making non-resuscitation decisions is a complicated process with both moral and legal implications [2], especially in out-of-hospital cardiac arrest, which is a global public health problem with low survival rates [3]. Approximately 60,000, 300,000 and 550,000 people per year experience cardiac arrest out-of-hospital in England, the United States and China respectively [4–6]. Where these patients were treated by emergency medical services, resuscitation was not attempted for approximately 20.7% in England, 10% in the United States and 75.5% in China [4, 5, 7].

Emergency medical services play a critical role in management of cardiac arrest out-of-hospital, and may explain some of the variation in outcomes [8]. The emergency medical system in China is part of the healthcare system and supported by government. This is different from many other countries. In China, ambulances are usually staffed by a physician, a nurse and a driver, and the physician is responsible for resuscitation for almost all cardiac arrests out-of-hospital. The ambulance crews can perform both basic and advanced life support including provision of intravenous fluids, endotracheal intubation, defibrillation, mechanical ventilation, and drug therapy. Ambulances are often called “moving intensive care units” in China. By law, non-physicians cannot make the decision not to attempt resuscitation or to terminate resuscitation either inside or out-of-hospital. However, there are no rules about not resuscitating or termination of resuscitation for emergency medical services providers.

The objective of this study was to investigate and understand the determinants of decisions not to attempt resuscitation on cardiac arrests out-of-hospital, to contribute to developing rules appropriate to China.

## Methods

### Study design and setting

We used a web platform (Questionnaire Star, https://www.wjx.cn/wjx/design/previewq.aspx?activity=106618688&s=1) for the survey. It was launched at 15:00 on February 5, 2021 and closed at 23:00 on March 6, 2021. We recruited 4318 participants using personal invitations sent through directors of emergency medical services across China and they all answered the questionnaire. The participants were all emergency physicians. We set an IP address that could only be used once, and the questionnaire could not be submitted if it was not filled in completely, to ensure validity and completeness. The time spent on each questionnaire was monitored automatically, and any questionnaires completed in fewer than 120 s were rejected as invalid. This study was approved by the Ethics Committee of Beijing Emergency Medical Center (2021–001) and written informed consent was waived. At the beginning of the survey, respondents were explicitly asked to give their consent to participate in the survey.

### Data collection

A questionnaire was developed for the study and used to collect demographic information and views on emergency work and cardiopulmonary resuscitation. It included questions on gender, age, nationality, religion, education, major, years of work experience, and views on cardiopulmonary resuscitation and when not to resuscitate. We determined participants’ geographic location using their IP addresses, and divided cities into first-, second-, and third-tier based on their level of economic development. Beijing, Shanghai, Guangzhou, and Shenzhen were first-tier cities. The 45 s-tier cities were mainly provincial capital cities and more economically developed cities. All other cities were third-tier [9].

### Views on not attempting resuscitation

We investigated why emergency physicians did not attempt cardiopulmonary resuscitation. We identified seven reasons from the literature and preliminary research, including decomposition/hypostasis/rigor mortis, massive injury, family members’ preferences, the patient’s expressed wishes, a medical history of serious illness, the patient’s age, and no bystander cardiopulmonary resuscitation. Respondents selected and sorted the factors they considered important. Each option was assigned a value of 7 to 1 points by level of importance. If an option was not selected, it was assigned a value of 0.

### Analysis

Continuous variables were expressed as mean and standard deviation, and categorical variables were shown as frequency and percentage in each category. Differences between different levels of cities were tested using the Kruskal–Wallis H-test. Differences in views on when not to resuscitate by categorical variables were tested using t-tests or one-way analysis of variance. Kruskal–Wallis H-tests were used when the variances were not uniform. Binary logistic regression analysis was used to identify the factors influencing how long after cardiac arrest was considered the cut-off for initiating cardiopulmonary resuscitation. All statistical analysis used SPSS version 22.0 (IBM Corp., Armonk, NY, USA), and *p* < 0.05 was considered to be statistically significant.

## Results

Of 4318 participants, 4289 (99.33%) met the criteria. They were from 31 provinces, autonomous regions and municipalities in mainland China. In total, 2264 (52.8%) were male and the largest proportion (1907, 44.5%) were between 30 and 40 years old. Most (3902, 91.0%) were of Han nationality; 2862 (66.7%) had an undergraduate degree; and 4080 (95.1%) had no religious belief. A total of 1760 (41.0%) worked in emergency medical services organisations at the prefecture level and 1767 (41.2%) at county level or below. Just under half (1946, 45.4%) had been working for less than 5 years; 1528 (35.6%) had majored in emergency medicine; and 2761 (64.4%) thought cardiopulmonary resuscitation was inappropriate if the cardiac arrest had happened more than 30 min before, and there had been no bystander cardiopulmonary resuscitation (see Table 1).

**Table 1:** Basic information of the investigator

	Total (*N* = 4289)	First-tier city (*N* = 1069)	Second-tier city (*N* = 1591)	Third-tier city (*N* = 1629)	Statistic value	P
Male(n,%)	2264(52.8)	625(58.5)	888(55.8)	751(46.1)	48.882	< 0.001
Age(n,%)					3.939	0.140
< 30 years	1185(27.6)	318(29.7)	413(26.0)	454(27.9)		
31–40 years	1907(44.5)	469(43.9)	756(47.5)	682(41.9)		
41–50 years	980(22.8)	227(21.2)	359(22.6)	394(24.2)		
51–60 years	217(5.1)	55(5.1)	63(4.0)	99(6.1)		
Han Nationality(n,%)	3902(91.0)	1013(94.8)	1450(91.1)	1439(88.3)	32.530	< 0.001
Education(n,%)					39.453	< 0.001
Master and above	286(6.7)	88(8.2)	138(8.7)	60(3.7)		
Undergraduate	2862(66.7)	674(63.0)	1100(69.1)	1088(66.8)		
Junior College and below	1141(26.6)	307(28.7)	353(22.2)	481(29.5)		
Religion(n,%)					6.932	0.031
No	4080(95.1)	1033(96.6)	1507(94.7)	1540(94.5)		
Buddhism	140(3.3)	20(1.9)	65(4.1)	55(3.4)		
Taoism	9(0.2)	1(0.1)	5(0.3)	3(0.2)		
Christian	26(0.6)	12(1.1)	9(0.6)	5(0.3)		
Islamic	34(0.8)	3(0.3)	5(0.3)	26(1.6)		
Level of city(n,%)					95.036	< 0.001
Provincial level	762(17.8)	333(31.2)	261(16.4)	168(10.3)		
Prefecture-level	1760(41.0)	319(29.8)	756(47.5)	685(42.1)		
County level and below	1767(41.2)	417(39.0)	574(36.1)	776(47.6)		
Work years(n,%)					14.171	0.001
< 5 years	1946(45.4)	547(51.2)	720(45.3)	679(41.7)		
5–10 years	1130(26.3)	238(22.3)	419(26.3)	473(29.0)		
11–20 years	976(22.8)	213(19.9)	386(24.3)	377(23.1)		
> 20 years	237(5.5)	71(6.6)	66(4.1)	100(6.1)		
Major(n,%)					28.972	< 0.001
Internal Medicine	971(22.6)	286(26.8)	381(23.9)	304(18.7)		
Surgery	499(11.6)	81(7.6)	203(12.8)	215(13.2)		
Gynaecology and Obstetrics	60(1.4)	12(1.1)	23(1.4)	25(1.5)		
Pediatrics	43(1.0)	4(0.4)	20(1.3)	19(1.2)		
Emergency Medicine	1528(35.6)	240(22.5)	588(37.0)	700(43.0)		
General practice	547(12.8)	215(20.1)	165(10.4)	167(10.3)		
Other	641(14.9)	231(21.6)	211(13.3)	199(12.2)		
Time for DNAR(n,%)					5.494	0.064
30 min	2761(64.4)	662(61.9)	1019(64.0)	1080(66.3)		
60 min	1528(35.6)	407(38.1)	572(36.0)	549(33.7)		

### Comparison between different levels of cities

In total, 1069 (24.9%) participants were from first-tier, 1591 (37.1%) from second-tier, and 1629 (38.0%) from third-tier cities. There were significant differences between levels of city in gender (H = 48.882, *p* < 0.001), nationality (H = 32.530, *p* < 0.001), education (H = 39.453, *p* < 0.001), religion (H = 6.932, *p* = 0.031), level of city (H = 95.036, *p* < 0.001), years of work experience (H = 14.171, *p* < 0.001), and major (H = 28.972, *p* < 0.001) (Table 1).

### Comparison of views on whether to resuscitate

**Fig. 1:**
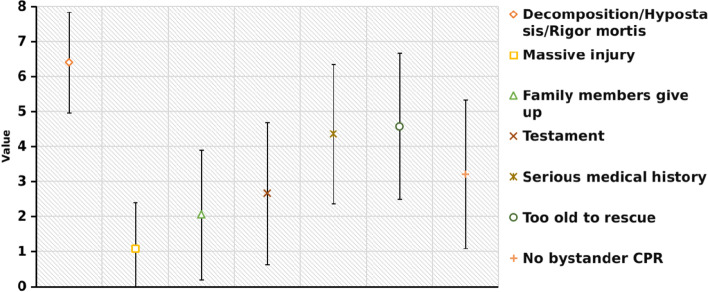
Reason of DNAR sequence diagram

The top reasons for not attempting resuscitation were decomposition/hypostasis/rigor mortis (6.39 ± 1.44 points), chosen by 4181 (97.5%), massive injury (4.57 ± 2.08 points), chosen by 3790 (88.4%) participants and family members’ preference (4.35 ± 1.98 points), chosen by 3843 (89.6%) participants. The fourth to seventh reasons in order of preference were the patient’s expressed views (3.20 ± 2.12 points, 3470 participants, 80.9%), medical history of serious illness (2.66 ± 2.03 points, 3096 participants, 72.2%), the patient’s age (2.05 ± 1.85 points, 2896 participants, 67.5%), and no bystander CPR (1.08 ± 1.32 points, 2713 participants, 63.3%) (Table 1 and Fig. 1).

For decomposition/hypostasis/rigor mortis, there were significant differences by gender, age, level and classification of city, and major (all *p* < 0.05) (Table 2). Compared with being male and of Han nationality, women and non-Han nationalities considered decomposition/hypostasis/rigor mortis less when deciding whether to attempt cardiopulmonary resuscitation. This factor was considered more important by those aged between 41 and 50 years than those in other age groups, and by those living in prefecture cities or second-tier cities. Those with a master’s degree and above were more likely to consider decomposition/hypostasis/rigor mortis, as were those with 11 to 20 years of work experience. Compared with other religions, those who professed Taoism also focused more on this reason (Supplementary table).

**Table 2:** Analysis of DNAR influencing factors

	Decomposition/Hypostasis/ Rigor mortis		No bystander CPR		Too old to rescue		Serious medical history		Family members give up		Massive injury		Will	
	Statistic value	P	Statistic value	P	Statistic value	P	Statistic value	P	Statistic value	P	Statistic value	P	Statistic value	P
Sex	2.604^a^	0.009	1.695^a^	0.090	2.747^a^	0.006	0.743^a^	0.457	3.023^a^	0.003	5.539^a^	< 0.001	4.729^a^	< 0.001
Age	14.423^b^	0.002	4.236^b^	0.237	1.903	0.127	48.758^b^	< 0.001	6.387	< 0.001	10.421^b^	0.015	23.311^b^	< 0.001
Nation	1.017^a^	0.309	1.921^a^	0.055	1.688^a^	0.091	0.148^a^	0.882	0.729^a^	0.466	0.168^a^	0.867	1.038^a^	0.299
Education	5.603^b^	0.061	4.255^b^	0.119	1.062^b^	0.588	3.749^b^	0.153	14.005^b^	0.001	27.221^b^	< 0.001	31.734^b^	< 0.001
Religion	0.336	0.854	1.577	0.178	0.954	0.432	0.141	0.967	0.272	0.896	7.998^b^	0.092	1.282^b^	0.865
Level of city	12.688^b^	0.002	2.621^b^	0.270	0.203^b^	0.903	7.562^b^	0.023	12.850^b^	0.002	6.670^b^	0.036	7.995^b^	0.018
Classification of city	40.208^b^	< 0.001	4.008^b^	0.135	1.977^b^	0.372	1.260^b^	0.533	32.467^b^	< 0.001	22.923^b^	< 0.001	30.477^b^	< 0.001
Work years	5.500^b^	0.139	2.930^b^	0.403	0.876	0.452	20.556	< 0.001	5.153	0.001	0.364	0.779	10.598	< 0.001
Major	21.618^b^	0.001	14.072^b^	0.029	14.405^b^	0.025	24.489^b^	< 0.001	1.806^b^	0.937	21.878^b^	0.001	9.549^b^	0.145

^a^Kruskal-Wallis H

^b^one-way analysis of variance

### Binary logistic regression analysis

**Fig. 2:**
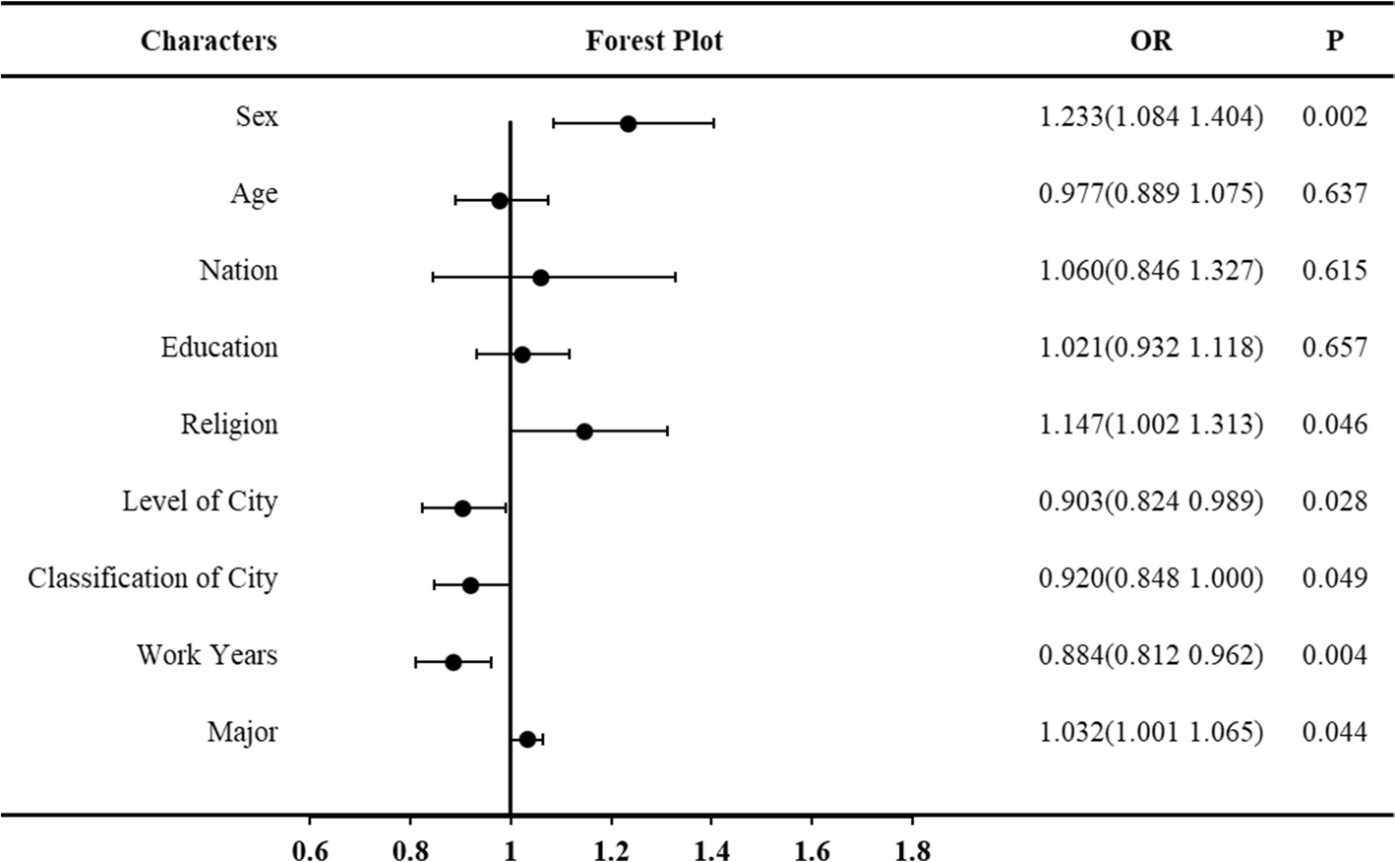
Influence factors of how long after OHCA does not initiate cardiopulmonary resuscitation

Age, gender, nationality, level of education, religion, level and classification of city, years of work experience, and major were included in the logistic regression analysis. Factor values are shown in Fig. 2. Gender (OR 1.233, *p* = 0.002), religion (OR 1.147, *p* = 0.046), level (OR 0.903, *p* = 0.028) and classification of city (OR 0.920, *p* = 0.049), years of work experience (OR 0.884, *p* = 0.004), and major (OR 1.032, *p* = 0.044) all influenced how long after cardiac arrest it was considered appropriate to initiate cardiopulmonary resuscitation.

## Discussion

The 2020 *American Heart Association Guidelines Update for Cardiopulmonary Resuscitation and Emergency Cardiovascular Care* provided only a few exceptions where withholding cardiopulmonary resuscitation would be considered appropriate [10]. The European Resuscitation Council Guidelines for Resuscitation 2015 also proposed that the decision not to start or to discontinue cardiopulmonary resuscitation is challenging out-of-hospital, because of the lack of information about patients’ wishes and values, comorbidities and baseline health status [11]. In China, there are no guidelines or rules for withholding cardiopulmonary resuscitation, and the decision mainly relies on the judgement of physicians. The behaviour and views of doctors and patients in China are affected by traditional Chinese culture, and are often very different from those held elsewhere. Emergency ambulances in China are also usually staffed by a physician, a nurse and a driver, and any resuscitation is usually conducted by the physician. American Heart Association and European Resuscitation Council guidelines for resuscitation are therefore not necessarily applicable in China. This study investigated views on when to resuscitate among emergency medical services physicians from around China, as a way to establish suitable rules for China. To our knowledge, this is the first survey in China of doctors’ views on when not to resuscitate. We found that the main factors influencing the decision not to perform cardiopulmonary resuscitation were decomposition/hypostasis/rigor mortis, massive injury, and family members’ preferences. Most emergency physicians (64.4%) would not perform cardiopulmonary resuscitation when cardiac arrest had occurred more than 30 min before and there had been no bystander cardiopulmonary resuscitation.

The top two reasons for not attempting cardiopulmonary resuscitation were decomposition/hypostasis/rigor mortis and massive injury. In total, 4181(97.5%) participants chose decomposition/hypostasis/rigor mortis, and 3226 (75.2%) placed it first. A total of 3790 (88.4%) participants chose massive injury, and 489 (11.4%) placed it first. This is consistent with the guidelines proposed by the American Heart Association and The European Resuscitation Council [12–14]. Age, gender, level and classification of city and major were all factors that affected these two choices. Men and emergency physicians aged 41–50 were more inclined to make those choices.

In addition to the obvious signs of death, most emergency physicians also tended not to perform cardiopulmonary resuscitation when cardiac arrest had happened more than 30 min before. Women were more likely than men not to attempt resuscitation when time since arrest was more than 60 min, as were emergency personnel with religious views. Physicians were less likely to attempt cardiopulmonary resuscitation more than 30 min after arrest with no bystander cardiopulmonary resuscitation when they worked for higher level emergency centres and in more prosperous cities. More experienced emergency personnel also thought that if cardiac arrest was more than 30 min ago, they should not give cardiopulmonary resuscitation. The guidelines for cardiopulmonary resuscitation in Europe, America and other countries suggest that cardiopulmonary resuscitation should not be initiated if the cardiac arrest was more than 15 min before. In China, physicians are therefore inclined to consider a longer time is more appropriate [15, 16], which is closely related to Chinese culture [17, 18].

Another important factor that affected views about whether to initiate cardiopulmonary resuscitation was the views of family members. This was ranked third in the survey. In China, emergency physicians will often respect the wishes of family members rather than consider the patient’s situation, such as their age [19, 20]. Even if a doctor’s professional judgement is that cardiopulmonary resuscitation is not appropriate, they may provide it if family members strongly request it. When family members explicitly ask them to stop, emergency personnel will not implement cardiopulmonary resuscitation and other related treatment methods under any circumstances.

Other factors like the patient’s expressed views, their medical history and age, and no bystander cardiopulmonary resuscitation also affected decisions about whether to perform cardiopulmonary resuscitation. These factors should also be considered as criteria for not providing cardiopulmonary resuscitation in the development of future guidelines for China.

This study had some limitations. First, the survey covered all provinces on the mainland, but the number of participants in some areas was relatively small. The survey may therefore not have fully captured the complexity of different responses to cardiac arrest out-of-hospital across China. Second, the order of the questions in the questionnaire was not randomised, so a learning effect in the course of responding to the questions cannot be excluded. Third, participants on web panels are more likely to be better educated, which could have affected the results. Last, we did not stratify by suspected mechanism or documented cardiac rhythm in our analysis due to inability to collect information. While case–control research is important direction for our follow-up, and we will definitely consider suspected mechanism and documented cardiac rhythm in our future study.

## Conclusions

Chinese emergency physicians have different perceptions from their foreign colleagues about when to resuscitate patients after cardiac arrest. The existing guidelines for resuscitation are therefore not suitable for China, and China-specific guidelines need to be established.

## Supplementary Information

**Additional file 1:** **Supplementary table.** Scores of factors affecting DNAR at different levels.

## Data Availability

http://doi.org/10.6084/m9.figshare.19076240

## Abbreviations

DNAR: Do not attempt resuscitation
OHCA: Out-of-hospital cardiac arrest
CPR: Cardiac Pulmonary Resuscitation
OR: Odds ratio

## References

[1] Perkins GD, Griffiths F, Slowther AM (2016). Do not attempt cardiopulmonary resuscitation decisions: an evidence synthesis.

[2] Fallahi M, Mahdavikian S, Abdi A (2018). Nurses and physicians' viewpoints about decision making of do not attempt resuscitation (DNAR). Multidiscip Respir Med.

[3] Berdowski J, Berg RA, Tijssen JG, Koster RW (2010). Global incidences of out-of-hospital cardiac arrest and survival rates: systematic review of 67 prospective studies. Resuscitation.

[4] Hawkes C, Booth S, Ji C (2017). Epidemiology and outcomes from out-of-hospital cardiac arrests in England. Resuscitation.

[5] Counts CR, Blackwood J, Winchell R (2021). Emergency Medical Services and Do Not Attempt Resuscitation directives among patients with out-of-hospital cardiac arrest. Resuscitation.

[6] Xu F, Zhang Y, Chen YG (2017). Cardiopulmonary resuscitation training in China:current situation and future development. JAMA Cardiol.

[7] Shao F, Li CS, Liang LR (2014). Outcome of out-of-hospital cardiac arrests in Beijing. China Resuscitation.

[8] Hazinski MF, Nolan JP, Aickin R (2015). Part 1: Executive summary: 2015 international consensus on cardiopulmonary resuscitation and emergency cardiovascular care science with treatment recommendations. Circulation.

[9] Jinhua Liu, Hongsheng Chen, Yang Chen (2018). Exploring the relationship between migrants' purchasing of commercial medical insurance and urbanisation in China. BMC Health Serv Res.

[10] Panchal AR, Bartos JA, Cabañas JG (2020). Part 3: Adult Basic and Advanced Life Support: 2020 American Heart Association Guidelines for Cardiopulmonary Resuscitation and Emergency Cardiovascular Care. J Circulation.

[11] MonsieursKoenraad G, Nolan Jerry P, Bossaert Leo L (2015). European Resuscitation Council Guidelines for Resuscitation 2015: Section 1. Executive summary. J Resuscitation.

[12] Aung Myat, Kyoung-Jun Song, Thomas Rea (2018). Out-of-hospital cardiac arrest: current concepts. Lancet.

[13] Ong MEH, Perkins GD, Cariou A (2018). Out-of-hospital cardiac arrest: prehospital management. Lancet.

[14] Tatsuma Fukuda, Naoko Ohashi, Takehiro Matsubara (2014). Applicability of the prehospital termination of resuscitation rule in an area dense with hospitals in Tokyo: a single-center, retrospective, observational study: is the pre hospital TOR rule applicable in Tokyo?. Am J Emerg Med.

[15] Jae Chol Y, Youn-Jung K, Shin A (2019). Factors for modifying the termination of resuscitation rule in out-of-hospital cardiac arrest. Am Heart J.

[16] Keita Shibahashi, Kazuhiro Sugiyama, Yuichi Hamabe (2018). A potential termination of resuscitation rule for EMS to implement in the field for out-of-hospital cardiac arrest: An observational cohort study. Resuscitation.

[17] Zhou G, Lu G, Shi O, Li X, Wang Z, Wang Y, Luo Q (2019). Willingness and obstacles of healthcare professionals to perform bystander cardiopulmonary resuscitation in China. Int Emerg Nurs.

[18] Zhu N, Chen Q, Jiang Z, Liao F, Kou B, Tang H, Zhou M (2019). A meta-analysis of the resuscitative effects of mechanical and manual chest compression in out-of-hospital cardiac arrest patients. Crit Care.

[19] Zhan L, Yang LJ, Huang Y, He Q, Liu GJ (2017). Continuous chest compression versus interrupted chest compression for cardiopulmonary resuscitation of non-asphyxial out-of-hospital cardiac arrest. Cochrane Database Syst Rev.

[20] Zhang L, Luo M, Myklebust H, Pan C, Wang L, Zhou Z, Yang Q, Lin Q, Zheng ZJ (2020). When dispatcher assistance is not saving lives: assessment of process compliance, barriers and outcomes in out-of-hospital cardiac arrest in a metropolitan city in China. Emerg Med J.

