# Supplementary material for: National survey of do not attempt resuscitation decisions on out-of-hospital cardiac arrest in China
Source: BMC Emerg Med. 2022 Feb 11;22:25. doi: 10.1186/s12873-022-00581-0 (PMC8832739; doi:10.1186/s12873-022-00581-0)
Supplement: Supplementary file 1 — Additional file 1: Supplementary table. Scores of factors affecting DNAR at different levels. [file 12873_2022_581_MOESM1_ESM.docx]

**Supplementary table Scores of factors affecting DNAR at different levels**

|  |  | Decomposition/Hypostasis/Rigor mortis | No bystander CPR | Too old to rescue | Serious medical history | Family members give up | Massive injury | Will |
| --- | --- | --- | --- | --- | --- | --- | --- | --- |
| Gender | Male | 6.44±1.35 | 1.04±1.30 | 1.98±1.83 | 2.68±2.04 | 4.26±1.97 | 4.73±2.06 | 3.05±2.09 |
|  | Female | 6.33±1.54 | 1.11±1.34 | 2.14±1.87 | 2.64±2.02 | 4.44±1.99 | 4.38±2.09 | 3.36±2.13 |
| Age | <30 years | 6.33±1.48 | 1.15±1.39 | 2.13±1.84 | 2.38±1.92 | 4.53±1.95 | 4.56±2.03 | 3.41±2.14 |
|  | 31-40 yers | 6.36±1.48 | 1.07±1.31 | 2.07±1.88 | 2.67±2.04 | 4.34±1.99 | 4.57±2.10 | 3.15±2.13 |
|  | 41-50 years | 6.52±1.31 | 1.00±1.20 | 1.95±1.78 | 2.95±2.08 | 4.18±1.93 | 4.68±2.04 | 3.10±2.04 |
|  | 51-60 years | 6.39±1.44 | 1.14±1.51 | 1.95±1.91 | 2.84±2.10 | 4.16±2.13 | 4.11±2.38 | 2.90±2.15 |
| Nationality | Han | 6.40±1.43 | 1.06±1.30 | 2.04±1.84 | 2.66±2.03 | 4.35±1.98 | 4.57±2.08 | 3.19±2.12 |
|  | Others | 6.32±1.53 | 1.21±1.50 | 2.20±1.89 | 2.68±2.04 | 4.28±1.98 | 4.58±2.08 | 3.30±2.07 |
| Education | Master and above | 6.50±1.18 | 0.89±0.94 | 1.98±1.74 | 2.68±1.98 | 4.86±1.44 | 4.64±1.84 | 3.79±2.01 |
|  | Undergraduate | 6.42±1.39 | 1.03±1.25 | 2.03±1.81 | 2.70±2.01 | 4.37±1.92 | 4.67±2.04 | 3.22±2.09 |
|  | Junior College and below | 6.27±1.61 | 1.23±1.55 | 2.13±1.97 | 2.56±2.10 | 4.16±2.20 | 4.29±2.21 | 2.99±2.18 |
| Religion | Religion | 6.39±1.44 | 1.08±1.32 | 2.06±1.84 | 2.66±2.03 | 4.35±1.98 | 4.57±2.08 | 3.20±2.11 |
|  | No | 6.28±1.65 | 0.91±1.22 | 1.81±1.89 | 2.60±2.16 | 4.21±2.13 | 4.31±2.26 | 3.07±2.28 |
|  | Buddhism | 6.67±0.71 | 0.56±0.53 | 1.89±2.09 | 2.78±2.68 | 4.00±1.94 | 5.22±1.20 | 2.78±2.33 |
|  | Taoism | 6.42±1.45 | 1.35±1.60 | 2.35±1.98 | 2.88±1.86 | 4.42±1.33 | 5.50±1.45 | 3.54±1.42 |
|  | Christian | 6.29±1.22 | 1.35±1.81 | 2.32±2.06 | 2.56±1.85 | 4.47±2.19 | 4.50±1.99 | 3.18±2.08 |
| Level of city | Provincial level | 6.29±1.48 | 1.05±1.24 | 2.06±1.78 | 2.77±1.98 | 4.54±1.82 | 4.74±2.00 | 3.38±2.05 |
|  | Prefecture-level | 6.47±1.29 | 1.02±1.27 | 2.05±1.84 | 2.71±2.03 | 4.44±1.91 | 4.54±2.06 | 3.20±2.09 |
|  | County level and below | 6.35±1.56 | 1.14±1.40 | 2.05±1.88 | 2.57±2.05 | 4.17±2.10 | 4.52±2.14 | 3.12±2.16 |
| Work Years | <5 years | 6.35±1.46 | 1.10±1.34 | 2.04±1.82 | 2.43±1.96 | 4.43±2.00 | 4.55±2.06 | 3.40±2.14 |
|  | 5-10 years | 6.40±1.46 | 1.09±1.35 | 2.10±1.87 | 2.69±2.02 | 4.40±1.91 | 4.61±2.06 | 3.04±2.11 |
|  | 11-20 years | 6.46±1.35 | 1.00±1.22 | 2.07±1.88 | 3.02±2.11 | 4.17±2.01 | 4.57±2.11 | 3.01±2.04 |
|  | >20 years | 6.35±1.59 | 1.15±1.41 | 1.90±1.81 | 2.96±2.09 | 4.11±1.99 | 4.48±2.23 | 3.09±2.11 |
| Major | Internal Medicine | 6.48±1.23 | 1.05±1.21 | 2.05±1.76 | 2.84±1.99 | 4.39±1.87 | 4.76±1.96 | 3.29±2.03 |
|  | Surgery | 6.51±1.31 | 1.03±1.24 | 1.93±1.77 | 2.61±2.10 | 4.30±1.99 | 4.65±2.14 | 3.10±2.01 |
|  | Gynaecology and Obstetrics | 6.18±1.78 | 1.28±1.51 | 2.10±1.85 | 2.72±2.00 | 4.27±2.13 | 4.78±1.87 | 3.40±1.98 |
|  | Pediatrics | 6.72±1.12 | 0.54±0.83 | 1.21±1.68 | 1.51±1.98 | 3.91±2.39 | 4.30±2.35 | 3.00±2.18 |
|  | Emergency Medicine | 6.40±1.44 | 1.09±1.39 | 2.05±1.91 | 2.65±2.04 | 4.37±2.00 | 4.43±2.11 | 3.12±2.17 |
|  | General practice | 6.31±1.57 | 1.12±1.35 | 2.13±1.83 | 2.67±2.00 | 4.35±1.96 | 4.67±2.03 | 3.16±2.14 |
|  | Other | 6.20±1.67 | 1.09±1.34 | 2.15±1.89 | 2.54±2.01 | 4.30±2.05 | 4.46±2.17 | 3.36±2.18 |
